# Draft Genome Sequences of Three Alkaliphilic *Bacillus* Strains, *Bacillus wakoensis* JCM 9140^T^, *Bacillus akibai* JCM 9157^T^, and *Bacillus hemicellulosilyticus* JCM 9152^T^

**DOI:** 10.1128/genomeA.01258-13

**Published:** 2014-01-30

**Authors:** Masahiro Yuki, Kenshiro Oshima, Wataru Suda, Yumi Oshida, Keiko Kitamura, Toshiya Iida, Masahira Hattori, Moriya Ohkuma

**Affiliations:** a Biomass Research Platform Team, RIKEN Biomass Engineering Program Cooperation Division, RIKEN Center for Sustainable Resource Science, Tsukuba, Ibaragi, Japan; b Center for Omics and Bioinformatics, Graduate School of Frontier Sciences, the University of Tokyo, Kashiwa, Chiba, Japan; c Japan Collection of Microorganisms/Microbe Division, RIKEN BioResource Center, Tsukuba, Ibaragi, Japan

## Abstract

Here, we report the draft genome sequences of the type strains of three cellulolytic or hemicellulolytic alkaliphilic *Bacillus* species: *Bacillus wakoensis*, *Bacillus akibai*, and *Bacillus hemicellulosilyticus*. The genome information for these three strains will be useful for studies of alkaliphilic *Bacillus* species, their evolution, and biotechnological applications for their enzymes.

## GENOME ANNOUNCEMENT

Alkaliphilic *Bacillus* strains have been isolated from various environments and have been well studied for their mechanisms of adaptation to alkaline conditions. These strains produce alkaline enzymes, such as protease, cyclomaltodextrin glucanotransferase, amylases, hemicellulase, and cellulase, which are useful for industry (1). *Bacillus wakoensis* strain JCM 9140^T^ and *Bacillus akibai* strain JCM 9157^T^ were isolated from soil as cellulolytic alkaliphiles, and their cellulose-degrading enzymes were characterized (2–5). A 16S rRNA gene sequence analysis indicated that the strains are closely related to each other and to *Bacillus krulwichiae* (5). *Bacillus hemicellulosilyticus* strain JCM 9152^T^, which is related to *Bacillus halodurans* and *Bacillus okuhidensis* (5), was isolated as a utilizer of alkaline hemicellulose-rich rayon waste and produces hemicellulases resistant to high pH conditions (6).

The genomes of *B. wakoensis* JCM 9140^T^, *B. akibai* JCM 9157^T^, and *B. hemicellulosilyticus* JCM 9152^T^ were sequenced using an Ion Torrent PGM system. The sequence reads totaling 621,952 for JCM 9140^T^, 523,952 for JCM 9157^T^, and 569,775 for JCM 9152^T^ were assembled using Newbler version 2.8 (Roche) into 187, 142, and 102 contigs, with N_50_ lengths of 51,263, 79,963, and 128,524 bp, respectively. These assemblies resulted in draft genome sequences of 4,530,938 bp for JCM 9140^T^, 4,749,369 bp for JCM 9157^T^, and 4,538,064 bp for JCM 9152^T^, with 24.6, 23.5, and 27.1× redundancies and G+C contents of 38.3, 37.0, and 38.2%, respectively. A total of 4,576, 4,711, and 4,345 protein-encoding genes and 108, 94, and 72 RNA-encoding sequences for JCM 9140^T^, JCM 9157^T^, and JCM 9152^T^, respectively, were identified using the RAST server (7) and the manual inspections detailed below.

RAST annotations and the subsequent CAZy database analyses (8) revealed that *B. akibai* JCM 9157^T^ and *B. hemicellulosilyticus* JCM 9152^T^ have in common various genes encoding xylanases classified in the glycoside hydrolase 8 (GH8), GH10, and GH43 families and β-xylosidases in the GH43 and GH52 families. *B. hemicellulosilyticus* JCM 9152^T^ has genes encoding cellulases of GH5 and GH16, whereas *B. akibai* JCM 9157^T^ has only that of GH16. Both strains have genes encoding β-glucosidase of GH3, and *B. hemicellulosilyticus* JCM 9152^T^ also has that of GH1. In the genome of *B. wakoensis* JCM 9140^T^, genes encoding β-xylanase of GH10, β-xylosidase of GH43, and β-glucosidase of GH1 were found. Although no cellulase gene was found in the RAST annotation, BLASTp analysis using the CAZy database showed that *B. wakoensis* JCM 9140^T^ has genes encoding cellulases of GH5 and GH9. These cellulolytic and hemicellulolytic enzymes have biotechnological potentials in various industries. Further comparative analyses on a genome scale will facilitate studies on the adaptation and evolution of alkaliphilic *Bacillus* species and their cellulolytic and hemicellulolytic enzymes.

### Nucleotide sequence accession numbers.

The genome sequences of *B. wakoensis* JCM 9140^T^, *B. akibai* JCM 9157^T^, and *B. hemicellulosilyticus* JCM 9152^T^ have been deposited in the DDBJ database under the accession no. BAUT01000001 to BAUT01000187, BAUV01000001 to BAUV01000142, and BAUU01000001 to BAUU01000102, respectively.
